# A new potential intervention strategy to promote the “body-mind-brain” health development of Chinese children and adolescents: a scoping review

**DOI:** 10.3389/fspor.2026.1862354

**Published:** 2026-08-27

**Authors:** Long Jin, Kai Qi, Zehao Zhang, Jia Yang, Yifan Shi, Aiguo Chen

**Affiliations:** 1 School of Physical Education and Sport, Henan University, Kaifeng, China; 2 School of Sport and Brain Health, Nanjing Sport Institute, Nanjing, China; 3 Gdansk University of Physical Education and Sport, Gdansk, Poland; 4 College of Physical Education, Yangzhou University, Yangzhou, China

**Keywords:** adolescents, body, brain, children, health, intervention strategy, mind, Shaolin martial arts

## Abstract

**Objective:**

This scoping review maps the extent and nature of literature on Shaolin martial arts (SMA) and examines how direct, contextual, and indirect evidence may inform hypotheses about body-mind-brain health in children and adolescents. It does not evaluate intervention effectiveness.

**Methods:**

Following PRISMA-ScR guidance, we searched PubMed, Web of Science, CNKI, and Wanfang Data from database inception to February 2026. Eligibility was defined by the population-concept-context framework. Records were classified as direct empirical evidence, contextual SMA literature, or indirect mechanistic evidence from related practices. Classical Chinese sources were used only to describe SMA principles and cultural context, not as evidence of health outcomes.

**Results:**

Twelve of the 13 included sources were contextual publications on SMA history, philosophy, education, or dissemination, and only one examined Shaolin boxing in children aged 8–10 years. The broader literature on martial arts, tai chi, qigong, meditation, and physical activity provides only indirect support for proposed physical, psychological, and cognitive mechanisms. No direct evidence currently establishes that SMA reduces anxiety or depression, improves executive function, alters brain activation, or induces neuroplasticity in children and adolescents.

**Conclusion:**

The current literature supports SMA as a culturally grounded candidate for future intervention development, but not as an empirically validated health intervention for children and adolescents. Feasibility studies and well-designed controlled trials with standardized SMA protocols and prespecified physical, mental-health, cognitive, and neurobiological outcomes are required before effectiveness claims can be made.

## 1 Introduction

In recent years, with rapid socioeconomic development and lifestyle changes in China, the health of children and adolescents has become an important public health concern. Chinese children and adolescents are increasingly facing multiple health challenges related to physical inactivity, prolonged sedentary behavior, psychological stress, and cognitive overload. At the physical level, insufficient physical activity and sedentary lifestyles have contributed to rising rates of obesity, declining physical fitness, and musculoskeletal health problems among Chinese youth (1–3). At the psychological level, increasing academic pressure and complex social environments have been associated with emotional problems such as anxiety, stress, and low self-esteem (4, 5). In addition, excessive use of digital devices and insufficient systematic cognitive stimulation may negatively affect attention, executive function, and cognitive development during adolescence, a critical period of brain maturation (6–9). Therefore, identifying effective intervention strategies to promote the physical, mental, and cognitive health of children and adolescents has become increasingly important in the Chinese context.

Physical activity has been widely recognized as an effective multidimensional intervention for promoting physical health, cognitive development, and psychological well-being in children and adolescents. Exercise interventions can improve cardiorespiratory fitness, musculoskeletal health, endurance, balance, and motor performance (10, 11). In addition, aerobic exercise has been shown to enhance executive function and academic performance through positive neurobiological adaptations (12, 13). Psychologically, moderate physical activity may alleviate depressive symptoms, improve self-esteem, and enhance social functioning (14, 15). However, although traditional exercise interventions have demonstrated substantial benefits for physical health, they may not fully address the psychological and emotional needs of children and adolescents. Consequently, mind–body exercise has attracted increasing attention as a more integrated health-promotion approach.

In the Chinese cultural context, traditional mind–body exercises have long been regarded as important approaches for promoting holistic health and self-cultivation. Practices such as tai chi and qigong combine physical movement with breathing regulation, mental concentration, and emotional adjustment, and have demonstrated positive effects on physical and psychological health (16–20). However, because some traditional mind–body exercises are relatively gentle and emphasize static meditation, they may be less attractive to highly active children and adolescents (21). Therefore, there is a growing need to explore mind–body interventions that better align with the behavioral and developmental characteristics of youth populations.

Shaolin martial arts (SMA), as a traditional Chinese mind-body practice, combine martial movements, physical conditioning, breathing regulation, and psychological cultivation. Descriptive and adult SMA studies document features such as strength, endurance, flexibility, coordination, concentration, emotional regulation, and mind-body integration (22, 23). These characteristics provide a rationale for considering SMA in youth research; however, they do not demonstrate health effects in children or adolescents. Accordingly, SMA is treated here as a culturally grounded candidate for intervention development rather than an established body-mind-brain intervention.

Direct intervention evidence on SMA in children and adolescents is scarce, and standardized youth protocols have not been established. This scoping review therefore maps the available SMA literature, separates direct youth-health evidence from contextual and indirect evidence, and identifies testable hypotheses and research gaps. The review does not estimate effect sizes or establish causal effectiveness.

## 2 Methods

This study followed the Preferred Reporting Items for Systematic Reviews and Meta-Analyses Extension for Scoping Reviews (PRISMA-ScR) guidelines (24) and the Arksey and O'Malley five-stage framework (25). A scoping approach was selected because the literature spans heterogeneous source types and contains little direct intervention evidence. The objectives were to map the available evidence, distinguish empirical health studies from contextual and mechanistic sources, and identify priorities for future youth-focused research, effectiveness was not evaluated.

### 2.1 Literature search and screening

We searched PubMed, Web of Science, CNKI, and Wanfang Data from database inception to February 2026. The search combined terms for Shaolin martial arts or Shaolin boxing with terms for children or adolescents and physical, psychological, cognitive, or brain-related outcomes; the database-specific search strings and yields are reported in Table 1. Two reviewers independently screened titles and abstracts and then assessed full texts. Disagreements were resolved by discussion and, when necessary, consultation with a third reviewer.

**Table 1:** Retrieval formula details.

Database	Query	Results
PubMed	(Martial Arts[Title/Abstract]) AND (Shaolin martial arts[Title/Abstract])	5
CNKI	(Topic: Shaolin Martial Arts) OR (Topic: Shaolin) AND (Topic: Children) OR (Topic: Teenagers)	5
Web of Science	(Shaolin martial arts) AND (Martial Arts)	108
Wanfang Data	(Shaolin Martial Arts) or (Shaolin) AND (Children and Adolescents)	2898

Eligibility was defined using a population-concept-context framework. We included peer-reviewed articles and academic theses in English or Chinese that addressed (1) populations identified by the original reports as children or adolescents, or (2) SMA principles, training, education, history, philosophy, or dissemination that were required to map the intervention concept. The original review protocol did not specify a numerical age threshold; therefore, age was charted as reported rather than assigned retrospectively. Sources were excluded if they were news items, non-academic commentary, duplicates, or did not provide substantive information about SMA. Health-effect claims required an empirical human study with a defined sample, exposure or intervention, and measured outcome. Adult, animal, general physical-activity, martial-arts, tai chi, qigong, meditation, and classical sources could inform contextual or mechanistic interpretation but were not treated as direct evidence of SMA health effects in children and adolescents. No publication-date restriction was applied. Restriction to English- and Chinese-language sources is acknowledged as a limitation.

Because SMA terminology and indexing vary across databases, we used database-specific subject headings and free-text terms. Broad retrieval in Wanfang Data accounted for most records, followed by eligibility screening against the same criteria. This strategy was designed to map a sparse field, it should not be interpreted as evidence that all retrieved records concerned youth health outcomes.

### 2.2 Data extraction and analysis

Two reviewers extracted data using a standardized form. For each included source, we recorded publication year, source type and design, population and age range, SMA content, comparator (if any), outcome domain and measure, principal finding, and limitations. Each source was also assigned to one evidence category: (A) direct empirical evidence on SMA and measured health or developmental outcomes in children or adolescents; (B) contextual SMA evidence on history, philosophy, education, dissemination, or intervention content; or (C) indirect mechanistic evidence from other populations or related practices. Categories B and C were used to formulate hypotheses and were not used to infer SMA effectiveness in the target population.

### 2.3 Quality assessment

Consistent with the purpose of a scoping review, we did not calculate a pooled risk-of-bias score or infer study quality from the journal in which a source appeared. Instead, we charted design features and evidentiary relevance, including whether a study directly examined SMA, enrolled children or adolescents, and measured a health or developmental outcome. These attributes determine how each source can contribute to the synthesis; journal prestige is not a proxy for study quality.

### 2.4 Data synthesis

The evidence was synthesized narratively by evidence category and outcome domain. Direct evidence (category A) was summarized first. Contextual evidence (category B) was used only to define SMA components and delivery settings, and indirect evidence (category C) was used only to identify plausible, testable mechanisms. We did not pool results because the evidence was sparse and heterogeneous, and we did not transfer effects observed for tai chi, qigong, general martial arts, physical activity, adults, or animals to SMA in children and adolescents.

### 2.5 Review framework

The review framework separates three evidentiary layers. Direct evidence requires SMA exposure in children or adolescents and a measured health or developmental outcome. Contextual evidence describes SMA history, philosophy, pedagogy, dissemination, or training content. Indirect evidence concerns related practices or other populations and can support only mechanistic plausibility. Conclusions are calibrated to these layers.

Accordingly, this review does not establish causal effects or definitive intervention outcomes. It develops a preliminary conceptual model and an empirical research agenda for testing whether defined SMA programs are feasible, acceptable, safe, and potentially beneficial for young people.

## 3 Results

### 3.1 Literature screening

**Figure 1:**
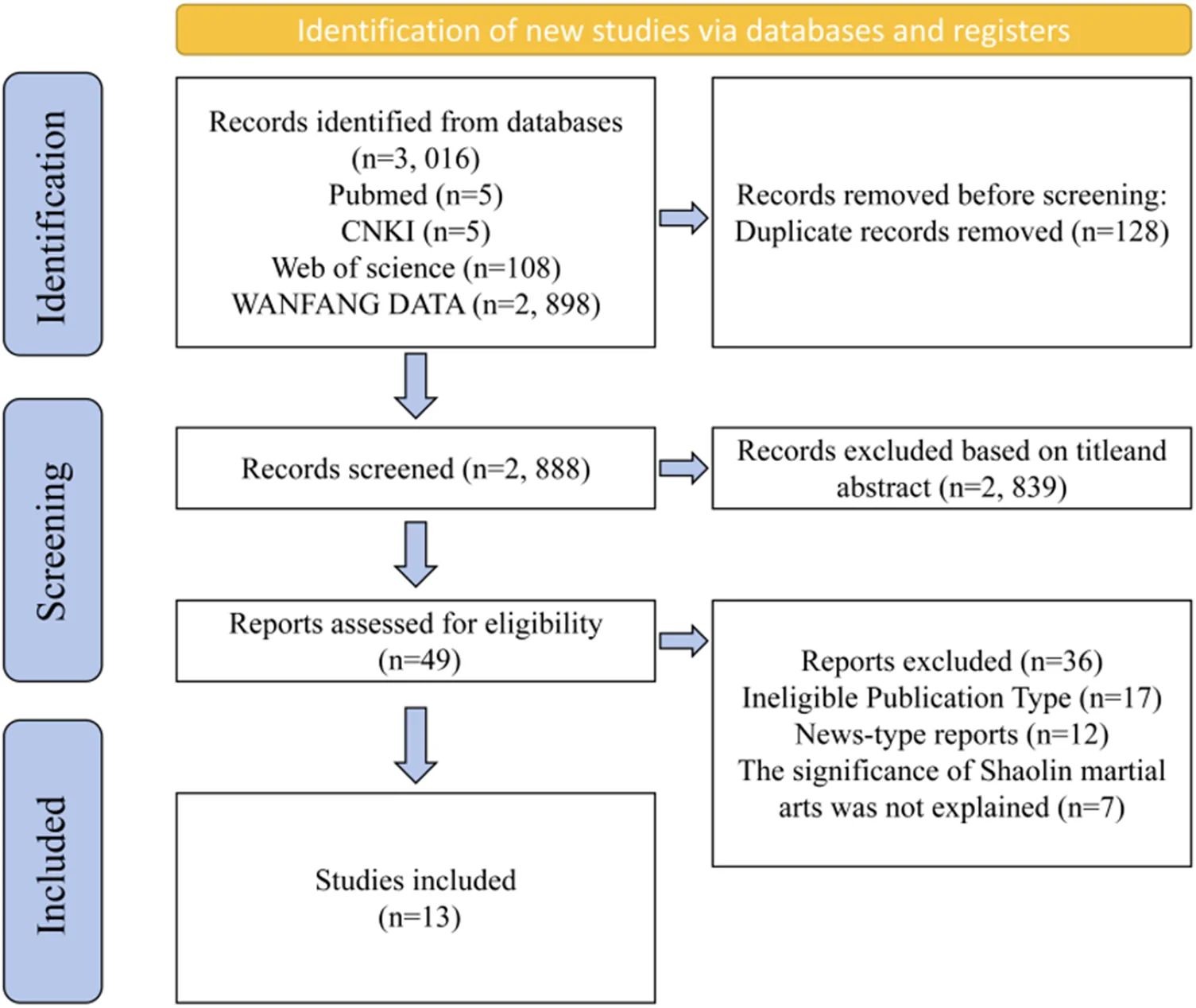
PRISMA flow diagram of study identification, screening, eligibility, and inclusion.

A total of 3,016 records were identified. After removing 128 duplicates, 2,888 records were screened; 49 full-text reports were assessed and 13 sources were included (Figure 1). Twelve sources were contextual accounts of SMA history, philosophy, education, or dissemination. One thesis examined Shaolin boxing as supplementary training in children aged 8–10 years. Thus, the included corpus was dominated by contextual evidence and did not provide a sufficient empirical basis for conclusions about health effectiveness.

### 3.2 Study characteristics

Table 2 charts the included sources by design, population, evidence category, relevance to youth health, and principal contribution. Evidence category indicates relevance to the review question and is not a study-quality score.

**Table 2:** Characteristics and evidentiary relevance of included sources.

Study	Design/source type	Population or context	Evidence category	Youth-health relevance	Principal contribution
Feng (2016) (26)	Descriptive literature analysis	International dissemination of SMA	B - contextual	None	Maps international communication pathways; no health outcome.
Hu & An (2014) (27)	Historical review	SMA in the Qing dynasty	B - contextual	None	Describes social, political, economic, and cultural context.
Liu (2012) (28)	Conceptual/descriptive analysis	SMA and Chan Buddhism	B - contextual	None	Describes philosophical characteristics and expression of SMA.
Li & Zhao (2012) (29)	Historical/conceptual analysis	Guangdong and Southern Shaolin martial arts	B - contextual	None	Discusses lineage and cultural relationships.
Li (2010) (30)	Conceptual analysis	Shaolin martial morality	B - contextual	None	Describes moral and cultural principles; no measured outcome.
Guo & Li (2017) (31)	Cultural analysis	Southern Shaolin intangible heritage	B - contextual	None	Explains cultural inheritance and collective memory.
Qiu (2006) (32)	Historical literature review	Developmental stages of SMA	B - contextual	None	Maps historical development and training traditions.
Liu & Wang (2004) (33)	Historical/educational review	SMA education	B - contextual	Indirect; no measured health outcome	Identifies educational functions and curriculum recommendations.
Wang (2011) (34)	Philosophical analysis	Integration of Chan and boxing	B - contextual	None	Describes moral and educational meanings of SMA.
Wan & Cai (2009) (35)	Philosophical analysis	SMA and Chan Buddhism	B - contextual	None	Describes meditation and mental-discipline concepts.
Chen (2019) (36)	Descriptive literature analysis	International promotion of SMA	B - contextual	None	Discusses dissemination strategy; no health outcome.
Shen & Yang (2025) (37)	Conceptual educational analysis	Secondary-school physical education	B - contextual	Indirect; no measured health outcome	Proposes school integration for cultural transmission.
Zhang (2021) (38)	Master's thesis; training study	Children aged 8–10 years; football training with Shaolin boxing	A - direct empirical	Physical-performance outcomes only	Reports agility, lower-limb explosive power, speed, and flexibility; does not address mental, cognitive, or brain outcomes.

Evidence category A: direct empirical youth-health evidence; B: contextual SMA evidence. Category describes relevance to the review question, not methodological quality. SMA, Shaolin martial arts; N/A, not applicable.

The evidence map shows a pronounced mismatch between the proposed health application and the available literature. Twelve included sources address the history, philosophy, morality, education, cultural transmission, or dissemination of SMA and therefore provide contextual information only. Zhang (2021) is the sole included source involving children aged 8−10 years and reports sport-performance outcomes when Shaolin boxing was used as supplementary football training; it does not establish broader physical-health, mental-health, cognitive, or neurobiological effects. Consequently, the included studies support definition of the intervention concept and research agenda, not claims of multidimensional health effectiveness.

For interpretive purposes, related literature cited elsewhere in the manuscript was classified as indirect evidence when it examined general martial arts, tai chi, qigong, meditation, physical activity, adults, animals, or neurostimulation rather than SMA in children or adolescents. Such studies were not counted among the 13 included SMA sources and were used only to identify candidate mechanisms or measures.

### 3.3 Direct evidence and hypotheses for physical and mental health

Direct evidence in the target population is limited to one thesis involving children aged 8–10 years in a football-training context (38). That study reported agility, lower-limb explosive power, speed, and flexibility outcomes, but the design details and generalizability available in the current evidence map are insufficient to attribute broad health benefits to SMA. The remaining included sources do not measure health outcomes in children or adolescents.

Contextual SMA sources describe conditioning, coordination, repetitive movement, breathing, meditation, discipline, and social practice. Research on general martial arts, physical activity, and Shaolin Neigong in other populations suggests candidate physical mechanisms (39–42), but these sources are indirect because the practice, population, or outcomes differ from the review question. They can inform intervention components and outcome selection, but cannot demonstrate that SMA improves fitness, balance, flexibility, or cardiopulmonary function in children and adolescents.

The same distinction applies to mental-health outcomes. Meditation, breathing, self-regulation, and group practice could plausibly influence stress, emotion regulation, self-confidence, or social functioning. However, the cited SMA-related sources either do not use a youth sample or do not measure anxiety, depression, resilience, or social adaptation (43–45). Therefore, statements about reduced anxiety or depression are hypotheses for future trials, not findings of this review.

**Figure 2:**
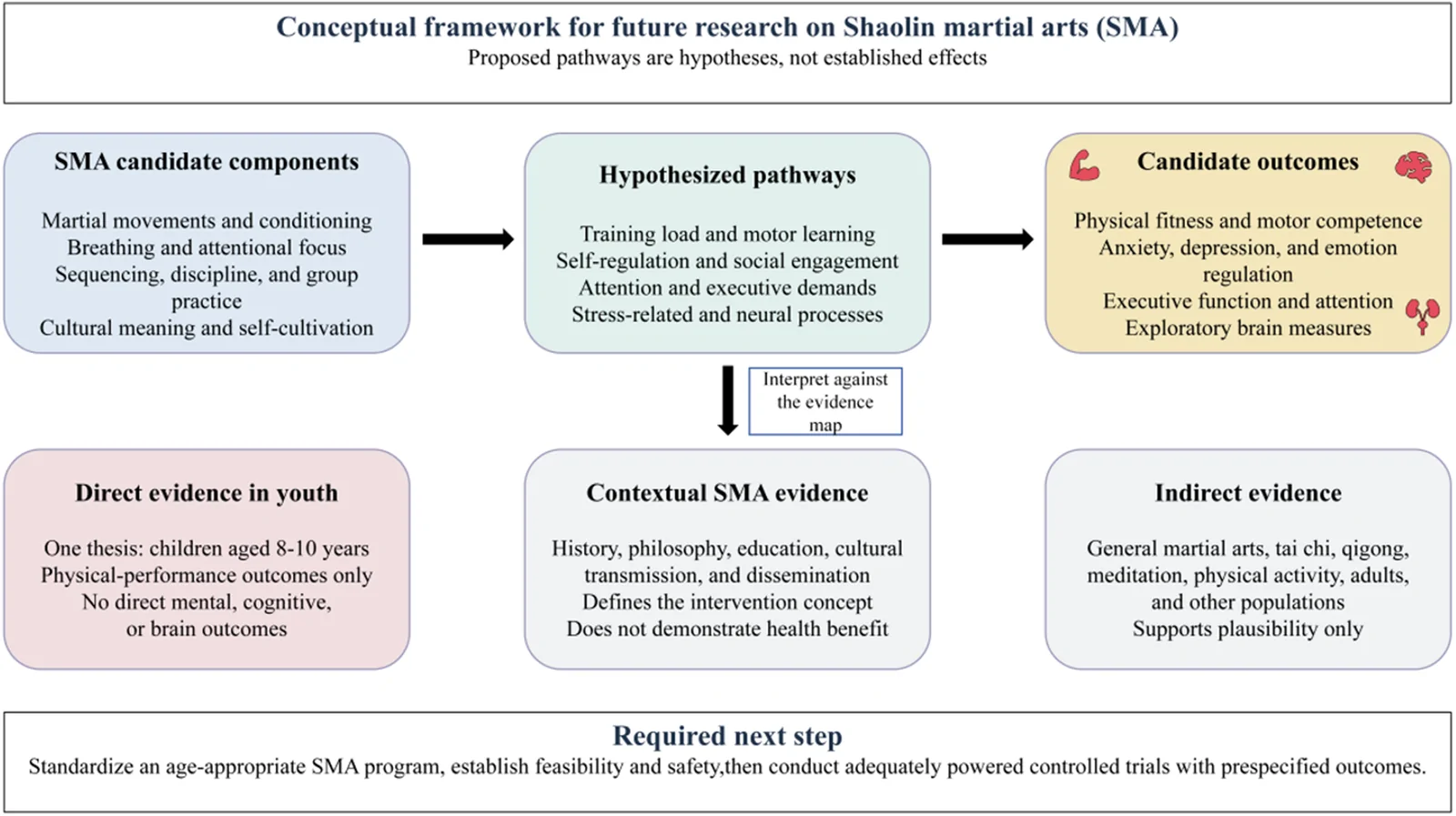
Conceptual framework of hypothesized SMA components and candidate body-mind-brain outcomes in children and adolescents. The proposed pathways are not established effects and require empirical testing.

The proposed physical and psychological pathways are summarized in Figure 2 as a conceptual model. All arrows represent hypotheses to be tested. Future feasibility studies should specify the SMA curriculum, instructor training, dose, safety monitoring, comparator, adherence, and validated physical and mental-health outcomes before efficacy trials are undertaken.

### 3.4 Indirect evidence and hypotheses for cognitive and brain health

SMA routines involve sequencing, visuospatial monitoring, sustained attention, inhibition, and rapid response. These task demands make executive function and attentional control reasonable candidate outcomes. Nevertheless, no included study directly measured cognitive or brain outcomes following SMA training in children or adolescents.

The previous version linked SMA to working memory, cognitive flexibility, and inhibitory control using animal exercise studies and non-SMA physical-activity research (46–48). These studies can illustrate general biological or task-related plausibility, but they cannot be used to infer a cognitive effect of SMA in young people. Future studies should use age-appropriate, prespecified behavioral measures and distinguish learning of the practiced routine from transfer to untrained cognitive tasks.

Evidence on brain activation is also indirect. Studies of acute exercise, tai chi, general martial arts, transcranial stimulation, and other populations report prefrontal or cognitive-control correlates (49–55), but these findings are not evidence that SMA changes brain activation or neuroplasticity in children and adolescents. Neuroimaging should therefore be considered an exploratory mechanistic outcome in future SMA trials, with appropriate control conditions, sample-size justification, and correction for multiple comparisons.

In summary, cognitive, emotional, and neurobiological pathways remain components of a conceptual framework rather than observed SMA effects. Figure 2 has been relabeled accordingly to prevent the proposed mechanisms from being interpreted as established benefits.

## 4 Discussion

### 4.1 Interpreting SMA as a potential body-mind-brain intervention

This scoping review identifies a culturally grounded rationale for developing and testing SMA programs, but it also demonstrates that the empirical foundation is very limited. The included corpus is dominated by historical, philosophical, educational, and dissemination research. Direct youth evidence consists of one context-specific thesis reporting sport-performance outcomes, while the proposed psychological, cognitive, and neural mechanisms are derived mainly from indirect literature. SMA should therefore be described as a candidate intervention concept, not as an effective or evidence-based intervention. The principal contribution of this review is the explicit evidence map and the set of testable hypotheses shown in Figure 2.

### 4.2 Relationship to related physical and mind-body practices

SMA combines vigorous movement, skill learning, breathing, attentional focus, discipline, and cultural meaning. These features differ from, but overlap with, general martial arts, tai chi, qigong, meditation, and conventional physical activity. Evidence from those practices may guide selection of components, comparators, and outcomes; it cannot be transferred directly to SMA because intervention content, dose, population, and context differ. Comparative advantages over conventional activities therefore remain hypothetical. Head-to-head feasibility and controlled studies are needed before claims of superiority or added value are warranted.

### 4.3 Research priorities for psychological, cognitive, and neural outcomes

Research should proceed in stages. First, investigators should define a reproducible, age-appropriate SMA program and assess feasibility, acceptability, adherence, instructor fidelity, and adverse events. Second, adequately powered controlled trials should prespecify validated physical, anxiety, depression, emotion-regulation, and executive-function outcomes. Only after behavioral effects are established should mechanistic studies use fNIRS, electroencephalography, or fMRI to test specific hypotheses about prefrontal activation, functional connectivity, or neural efficiency. Such studies require appropriate active controls, longitudinal designs, transparent analysis plans, and cautious interpretation because current neuroimaging evidence comes from non-SMA practices or populations (52–55).

### 4.4 Policy and educational practice implications

The current evidence does not justify routine implementation of SMA as a health intervention. Schools and community organizations may consider carefully monitored pilot programs for cultural education and physical activity, but health claims should not be made until benefits and safety are demonstrated. Policy recommendations should await standardized protocols, feasibility data, and controlled evidence. In the interim, this review can guide co-design of pilot programs with young people, families, educators, clinicians, and qualified SMA instructors.

### 4.5 Limitations

Because intervention-based evidence and standardized youth protocols are lacking, this review maps a preliminary concept rather than demonstrating effectiveness.

Several limitations should be acknowledged. First, the review was restricted to English- and Chinese-language sources and was dominated by Chinese publications, creating language and selection bias and limiting international generalizability. Second, broad retrieval and inconsistent indexing generated a large screening set, while only 13 heterogeneous sources met the review criteria. Third, most included sources were non-empirical or contextual, and the one youth study was context-specific; no pooled or causal inference was therefore possible. Fourth, indirect evidence from related practices and other populations was used only to develop hypotheses, but conceptual transfer may still oversimplify important differences in content, dose, and context. Fifth, no formal risk-of-bias appraisal was undertaken; evidence categories indicate relevance, not methodological quality.

Classical Chinese and descriptive sources were consulted to define historical, philosophical, and training features of SMA. They cannot substantiate health outcomes. The absence of standardized intervention protocols, direct measures of anxiety or depression, cognitive testing, and youth neuroimaging evidence further limits interpretation.

Overall, these limitations should be considered when interpreting the findings of this study.

## 5 Conclusion

This scoping review maps SMA as a culturally grounded candidate for future intervention development in children and adolescents. The available corpus is dominated by contextual literature, with only one context-specific youth study and no direct evidence for mental-health, executive-function, brain-activation, or neuroplasticity effects. Evidence from related practices supports plausible research questions but cannot be transferred as proof of SMA benefits. Standardized protocols, feasibility work, and well-designed controlled trials are required before SMA can be recommended as an effective body-mind-brain health intervention.

## Data Availability

The original contributions presented in the study are included in the article/Supplementary Material, further inquiries can be directed to the corresponding author.
